# Genetic Structure and Conservation Management of Endemic *Salix kusanoi* in Fragmented Habitats of Taiwan

**DOI:** 10.3390/plants14071080

**Published:** 2025-04-01

**Authors:** Piumi Chathurika Palangasinghe, Ya-Zhu Ko, Tsai-Wen Hsu, Manupa Pabasara Wickramasinghe, Huei-Chuan Shih, Meng-Shin Shiao, Yu-Chung Chiang

**Affiliations:** 1 Department of Biological Sciences, National Sun Yat-sen University, 70 Lienhai Road, Kaohsiung 804, Taiwan; piumipalangasinghe@gmail.com (P.C.P.); abscl77512@gmail.com (Y.-Z.K.); manupa1993@gmail.com (M.P.W.); 2 Taiwan Biodiversity Research Institute, Nantou 552, Taiwan; twhsu@tbri.gov.tw; 3 Department of Nursing, Meiho University, Pingtung 912, Taiwan; x00002213@meiho.edu.tw; 4 Research Laboratory Section, Offices of Health Science Research, Faculty of Medicine Ramathibodi Hospital, Mahidol University, Nakhon Pathom 10400, Thailand; 5 Department of Biomedical Science and Environment Biology, Kaohsiung Medical University, Kaohsiung 807, Taiwan

**Keywords:** genetic structure, *Salix kusanoi*, fragmented habitats, conservation management

## Abstract

*Salix kusanoi* is an endangered riparian tree species endemic to Taiwan. This study aimed to evaluate the genetic diversity and population structure across eight fragmented populations employing 33 microsatellite loci. The findings revealed moderate genetic diversity (mean *A_E_* = 3.85, *H_O_* = 0.22) and significant deviations from the Hardy–Weinberg equilibrium. This indicated an evolutionary pressure, such as genetic drift and inbreeding. The Analysis of Molecular Variance (AMOVA) demonstrated evident genetic differentiation among populations (*F_ST_* = 0.30). Principal Coordinates Analysis (PCoA) and Bayesian clustering (STRUCTURE) described distinct regional genetic patterns, with K = 5 providing a robust context for understanding localized genetic variation. Conservation interventions, including targeted in situ conservation for genetically unique populations (SBF) and genetic rescue strategies for genetically underprivileged populations (NW and NT), are proposed to safeguard the genetic integrity and adaptive potential of *S. kusanoi*.

## 1. Introduction

Habitat fragmentation and climate change pose significant threats to plant biodiversity worldwide, with approximately 40% of plant species currently at risk of extinction [1]. These anthropogenic pressures have intensified in recent decades, leading to the decline or disappearance of numerous taxa and populations. The destruction of fragile ecosystems is a primary driver of species extinction [2,3]. To address these challenges and develop effective conservation strategies for rare species, it is crucial to utilize genetic data prior to implementing on-the-ground conservation efforts. This approach helps minimize potential risks associated with inbreeding or outbreeding depression.

The genus *Salix* is recognized as a pioneer species, often being the first woody vegetation to establish itself in various ecosystems, including riparian zones, low-lying wetland areas, and alpine environments. This underscores the ecological importance of *Salix* in initiating vegetation succession in diverse habitats [4]. However, the conservation of *Salix* genetic resources faces significant challenges as some *Salix* species face habitat loss, particularly the destruction of wetlands resulting from industrial expansion and urbanization [5,6,7].

*Salix kusanoi* (Hayata), commonly known as the Shui-sheh willow or Kosano willow, is an endangered, endemic riparian tree species native to Taiwan. It was first discovered along the shores of Sun Moon Lake near Shuishe Village. This dioecious, catkin-bearing perennial shrub relies on both wind (anemophily) and insect (entomophily) pollination [8]. Thriving in wetlands and ponds, it can grow up to 8 m tall but remains confined to a narrow habitat range. However, human activities and environmental changes, including the construction of reservoirs and the resulting rise in water levels, have led to a significant decline in its populations [9]. These challenges emphasize the species’ ecological dependence on fragile riparian ecosystems and underscore the urgent need for targeted conservation efforts to ensure its survival.

As of now, the species is sparsely distributed in Nantou, Yilan, and Pingtung areas in Taiwan, ranging from lowlands to mountainous regions up to 1500 m in elevation [10]. The plants thrive in riparian zones and areas adjacent to streams and rivers. They are, thus, playing a crucial role in preventing soil erosion and maintaining the structural integrity of riverbanks. According to the “Red List of Vascular Plants of Taiwan” [11] and the IUCN Red List [12], *S. kusanoi* has been assessed as endangered based on its limited geographic range. Therefore, the urgency for conservation is particularly evident to mitigate the negative genetic effects associated with small, isolated populations and to reduce the risk of outbreeding depression during restoration efforts; it is crucial to explore the genetic diversity and connectivity of the remaining fragmented *S. kusanoi* populations. Genetic diversity is crucial for population dynamics and evolutionary potential [13]. Diverse plant species enhance overall biodiversity and ecosystem resilience, particularly important for adapting to climate change. Despite its importance, there is a notable scarcity of research examining the genetic structure and diversity of the fragmented *S. kusanoi* populations in Taiwan. Obtaining comprehensive genetic data on *S. kusanoi* would offer vital insights for conservation biologists and geneticists and for the implementation of effective breeding programs.

Molecular techniques have emerged as crucial tools in advancing both in situ and ex situ conservation strategies for Salix species. Studies of genetic diversity, divergence, and gene flow among populations have been conducted on several Salix species using various genetic markers. Molecular markers, such as restriction fragment length polymorphisms (RFLPs), Random Amplified Polymorphic DNA (RAPD), amplified fragment length polymorphisms (AFLPs), Inter Simple Sequence Repeats (ISSRs, or microsatellites), and single nucleotide polymorphisms (SNPs), have been widely used in Salix phylogenetic relationships and genetic diversity analysis [14,15,16,17,18,19,20,21,22,23,24]. Microsatellite markers have been widely used and are considered ideal for studying genetic diversity and population structure in many willow species [17,25,26,27,28,29,30,31,32,33,34]. Despite this, there has not yet been a comprehensive study examining the population-level genetic variation and genetic structure in *S. kusanoi* across diverse ecosystems utilizing microsatellite markers.

Based on the literature, studies have shown that river systems and geographical factors significantly influence the genetic structure of Salix species [19,20,28]. Additionally, this endangered species faces substantial habitat loss and fragmentation due to factors such as urban development, agricultural expansion, and alterations to river flow dynamics. Therefore, we hypothesized that geographic isolation, rather than habitat fragmentation, has significantly influenced the genetic diversity and population structures of this endemic species, resulting in geographically correlated genetic patterns. This study aimed to comprehensively assess the genetic diversity and population structures of *S. kusanoi* in Taiwan using polymorphic microsatellite markers to investigate the impact of habitat fragmentation. The objectives of this study were as follows: (1) Evaluate the level of genetic diversity and population structures of *S. kusanoi*. (2) Identify the spatial genetic pattern of *S. kusanoi*. (3) Provide evidence-based recommendations for conservation strategies based on the genetic diversity and population structures, ensuring the genetic integrity and long-term conservation of the endemic species.

## 2. Results

### 2.1. Genetic Diversity of Salix kusanoi Populations in Taiwan

The genetic diversity of *S. kusanoi* was evaluated based on the distribution of alleles and the levels of heterozygosity of the loci across eight populations (Supplementary Table S3). The population in the Shuanglian Pond (SB, 20 individuals) had the highest diversity at loci AG-44 and AG-87, each with eight alleles, while AG-25 and AG-117 were monomorphic. The effective number of alleles (*A_E_*) ranged widely, peaking at AG-87, which also displayed the highest observed heterozygosity (*H_O_* = 0.800). In the population in Zhongming Village (NC, 11 individuals), the most allelic diversity was observed at AG-18, while AG-45 showed the greatest effective alleles and highest *H_O_* (0.636). The population in the Wujie Reservoir (NW, nine individuals) also showed diversity peaks at AG-140 with six alleles, with high *A_E_* and *H_O_* values concentrated at this locus. The population in the Toushe Basin (NT, six individuals) showed the highest allelic and effective allele counts at the AC-2 locus, while *H_O_* values were low at other loci. The population in the Dongyuan Wetlands (PM, 28 individuals) showed the most pronounced diversity at locus AG-45 with 17 alleles, a high AE value (8.430), and the highest *H_O_*. The other three populations, i.e., in Floating Island at Shuanglian Pond, Mysterious Lake, and Sheng Lake, exhibited similar patterns with multiple loci displaying high allelic diversity, and each recorded the maximum *H_O_* of 1.000 at several loci. To be noted, a total of 27 alleles were observed at locus AG-45, making it the locus with the highest level of polymorphism. Across all populations, significant deviations from the Hardy–Weinberg equilibrium (*HWE*) were observed, especially at loci with high allelic variation (Supplementary Table S3).

### 2.2. Genetic Differentiation Among Salix kusanoi Populations

The Analysis of Molecular Variance (AMOVA) was performed to partition the genetic differentiation of the species in the three geographic regions located in northeast (Yilan), central (Nantou), and southern Taiwan (Pingtung), which represented the natural geographic isolations of plant species (Table 1). To be noted, there is a floating island in Shuanglian Lake, and there are individuals of *S. kusanoi* from both locations, that is, in the lake (SB) and on the floating island (SBF). The genetic differentiation between the populations in the lake and on the island provides evidence of the impact of geographic fragmentation on the population structures. Across geographic regions, the genetic differentiation among regions accounted for only 15% of the total genetic variation (*F_ST_* = 0.15, *p* < 0.001). The highest variation was observed among populations (49%, *F_IS_* = 0.58, *p* < 0.001). The remaining 36% of the variation was found within populations (*F_IT_* = 0.64, *p* < 0.001).

**Figure 1:**
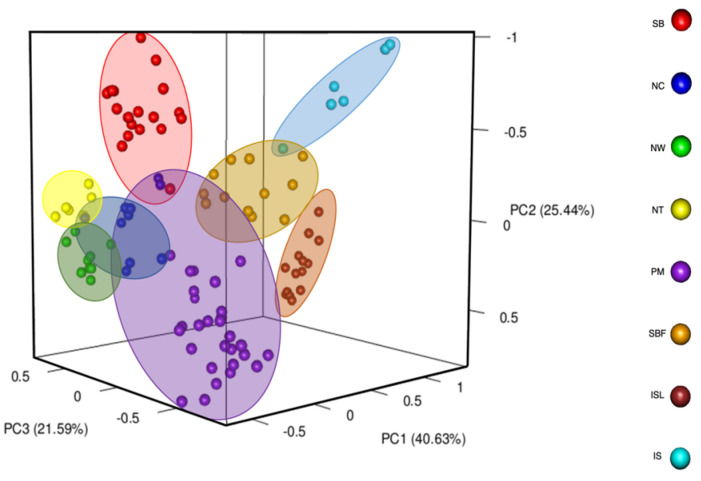
Principal Coordinates Analysis (PCoA) of genetic diversity of *Salix kusanoi* populations. Different colors indicate distinct populations, and proximity of points reflects genetic similarity. Percentage of variance explained by each principal coordinate axis is shown in parentheses.

Principal Coordinates Analysis (PCoA) showed that the primary principle accounted for 87.65% of the total genetic variation (Figure 1). The PCoA results reveal clear genetic structuring among populations. The SB population from Shuanglianpi and the NW population from Renai, Nantou, are positioned at opposite ends of the plot. The PM population from Mudan, Pingtung, clusters separately from most other populations, while the IS and ISL populations from Suao and the Mysterious Lake in Yilan are positioned closely together. The NC population from Zhongming village in Yuchi, Nantou, appears more widely spread across the axes and the NT population forms a relatively compact cluster.

### 2.3. Population Genetic Structure of Salix kusanoi Across Eight Populations

**Figure 2:**
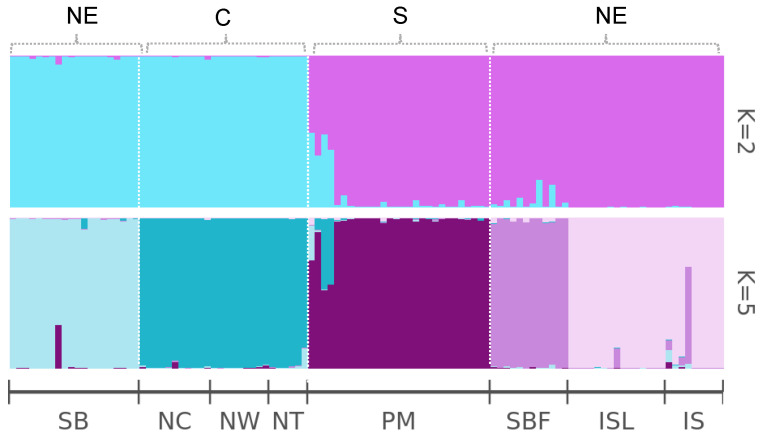
*K* = 2 and *K* = 5 cluster STRUCTURE ancestral proportion bar chart. Each population is represented as segment, divided vertically by different colors, representing proportion of ancestors estimated by individual in each cluster.

The Bayesian cluster analysis identified Δ*K* = 2 as the optimal *K* value (Figure 2). However, *K* = 5 was also considered biologically significant, as it was identified by Evanno’s method [35] as the second-best *K* value, with the second lowest standard deviation for the Logarithm of the Probability of the Data Given *K* (stdev LnP(*K*)). Using *K* = 2, genetic variations were grouped into two clusters based on geographic location: northeast Taiwan (pink) and central–southern Taiwan (blue), except for the SB population. Although the SB population is geographically situated in northeast Taiwan, its genetic composition is more closely related to populations in middle–southern Taiwan. A similar clustering pattern for SB was also observed when analyzed with *K* = 5. We will discuss the possibilities of the observation in later paragraphs.

**Figure 3:**
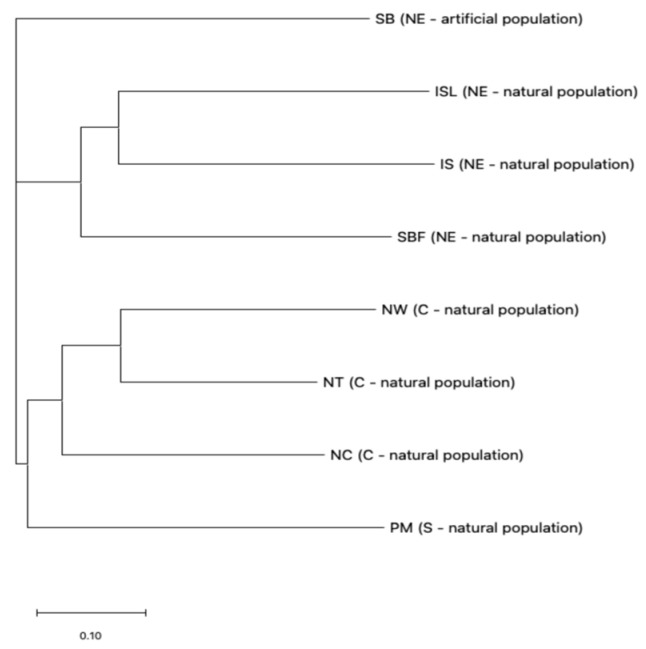
Neighbor-Joining dendrogram based on Nei’s genetic distance among 8 populations of *Salix kusanoi*. Geographical region natural/artificial nature of population is given in parentheses, and scale is given in bottom.

The genetic distance (GD) between the eight populations of *S. kusanoi* ranged from 0.4174 to 0.7509, with an average of 0.0639 (Table 2). The GD between the populations of NT and NW was the smallest (0.4174) as they were both from Nantou County. In contrast, even though SB and IS were both from Yilan County, they showed the greatest GD (0.07509) among the comparisons between populations. The populations of NT and PM were intermediate in their genetic relationships, showing moderate GD values. Clustering analysis showed that the populations of SBF, ISL, and IS formed a cluster, while the populations of NC and NW formed another cluster. However, the SB population was clustered separately, without clustering with any of the other populations (Figure 3).

### 2.4. Genetic Barrier Analysis and Migration

**Figure 4:**
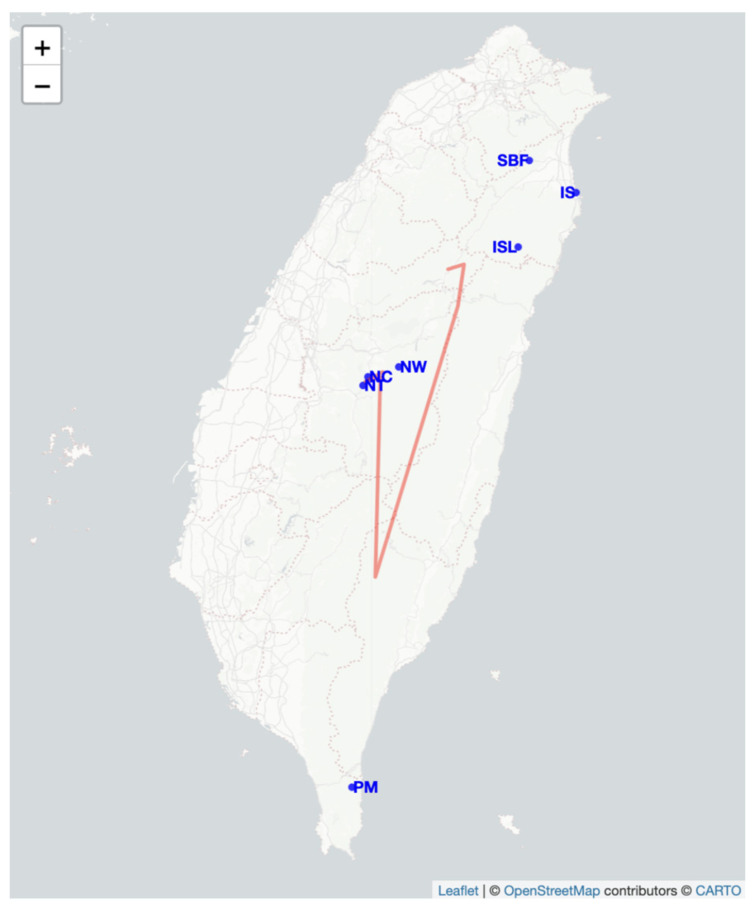
The significant genetic barrier identified among populations using Monmonier’s Algorithm. The red solid lines represent the barrier paths, and the populations are labeled as blue points.

We analyzed genetic barriers between populations using Monmonier algorithm implemented in the adegenet package in R (version 2.1.11) which revealed significant genetic discontinuities isolating the SBF, ISL, and IS populations from the others (Figure 4). These barriers indicate restricted gene flow and strong genetic isolation for these populations. The SBF population, located on the Shuanglianpi floating island, displayed particularly pronounced isolation, as evidenced by prominent barriers surrounding it. In contrast, populations such as NT, NW, and NC exhibited fewer barriers, suggesting relatively higher levels of genetic connectivity. The absence of significant barriers near PM implies reduced genetic differentiation or increased gene flow in this region (Figure 4). Notably, the cultivated SB population was excluded from this analysis, as its artificial establishment does not reflect natural genetic processes and could confound the identification of true genetic barriers.

The migration matrix revealed that most populations exhibited high self-recruitment rates, indicating strong population stability. This may also suggest a potential for high levels of inbreeding within these populations. (Table 3). PM and ISL demonstrated the highest self-recruitment rates of 94.24% and 90.95%, respectively, suggesting minimal genetic exchange with other populations. Conversely, NT showed the lowest self-recruitment rate at 84.63%. Notable migration events were observed even at low levels. Among the populations of central Taiwan, NC contributed migrants to NW and NT at rates of 1.87% and 1.85%, respectively. Additionally, NW contributed 2.60% of migrants to NT and 2.12% to ISL. Overall, off-diagonal migration rates were generally below 3%.

## 3. Discussion

The panel of 33 microsatellite markers used in this study provided informative data to characterize *S. kusanoi* population structures and genetic variations in eight populations in Taiwan. A moderate to high level of polymorphism indicates that they are effective in revealing the genetic variation in *S. kusanoi*. An average number of 8.242 observed alleles (*Na*) and 3.852 effective alleles (*Ne*) per locus within 110 individuals was observed. Additionally, all eight populations showed lower values of observed heterozygosity (*H_O_* = 0.22) when compared with the other *Salix* species.

From the literature based on the studies using microsatellites, species in the genus usually showed significantly high heterozygosity as they are outcrossing species. For example, *Salix viminalis* in the Ergun and West Liao basin showed a heterozygosity of 0.638 [36]. An average heterozygosity of 0.41 was also observed for the *Salix caprea L*. in Ireland [23]. The observed heterozygosity was high and comparable to the well-documented genetic diversity within the family Salicaceae [37,38]. A deficit of heterozygotes has previously been reported in several Salicaceae species, including *S. lapponum* and *S. lanata* [39], *S. daphnoides* [40], *Populus alba* [41], and *P. davidiana* [42]. The moderate genetic diversity observed in *S. kusanoi* may be attributed to anthropogenic activities impacting its habitats, as well as other environmental pressures occurring in the habitats as well as the high inbreeding rate in some of the populations revealed by this study [9,40].

To identify the factors influencing the current distribution and genetic variation in *S. kusanoi* in Taiwan, the spatial scale of the genetic diversity was examined using multiple approaches. The overall genetic differentiation among populations was significant. The differentiation between sampled sites, measured by *F_ST_*, was statistically significant. The among-population variation accounted for 30% of the total genetic variation, while 34% was attributed to variation within populations (Table 2). Given that the SB population represents a cultivated population, we recalculated *F_ST_* after its removal. The results showed an *F_ST_* of 0.18 among geographical regions and 0.40 among populations, indicating a moderate to high level of genetic differentiation. The comparison between these *F_ST_* values suggests that removing the SB population did not substantially alter the overall genetic structure, reinforcing the idea that these populations are relatively isolated and exhibit distinct genetic compositions. According to [43], *F_ST_* values between 0.15 and 0.25 indicate moderate genetic differentiation, while values above 0.25 indicate high differentiation. Our results suggest substantial genetic structuring among *S. kusanoi* populations, with limited gene flow between them.

As AMOVA was conducted across three geographical regions (Yilan, Nantou, and Pingtung), only 15% of the variation was explained by the differences among regions. These findings align with other studies on Salicaceae, which generally report low to moderate differentiation [44,45].

The AMOVA results were further supported by PCoA, where the populations from Yilan, Nantou, and Pingtung were clustered distantly along the PC1 axis as they are geographically isolated. The SBF population was grouped into a distinct genetic cluster separate from the SB and IS populations, despite all originating from Yilan. Notably, SBF was located on the floating island of the lake, which is also the origin of the SB population. This might be since the unique ecological form of the floating island allowed the population to be independently differentiated [46]. The results were further proved by the unweighted Neighbor-Joining tree, where SBF and SB populations from Shuanglianpi were clustered separately, and IS and ISL populations were sister groups.

The STRUCTURE analysis revealed that the SB population in northeast Taiwan is genetically similar to the populations from central and southern Taiwan (Nantou and Pingtung, respectively). This might result from human interreference with the cross-plantation of the same species from the Toushe basin in Nantou to the Shuanglianpi region. Therefore, the SB population is genetically different from the SBF population located on the floating island. The STRUCTURE analysis with *K* = 5, grouped the population into five clusters following the same pattern as the PCoA and NJ tree.

The barrier analysis using the Monmonier Algorithm identified the Central Mountain Range (CMR) as a prominent geographic barrier contributing to significant genetic discontinuities among the studied populations. The detected paths of maximum genetic differences spanned regions corresponding to populations such as IS, SBF, and NC, located near or across the CMR. Despite some geographic proximity among these populations, the high genetic differentiation values (e.g., 7.92) indicate restricted gene flow, likely influenced by the rugged terrain and steep elevation gradients of the CMR. This supports the hypothesis that topographic barriers, rather than geographic distance alone, drive genetic isolation in this region. Furthermore, the substantial variation in genetic differences between paths (e.g., 0.792 vs. 0.538) highlights the impact of localized ecological processes, such as habitat fragmentation, environmental gradients, and historical isolation, on the genetic structure of populations. These findings align with previous studies emphasizing the ecological significance of Taiwan’s mountainous landscape as a natural divider of biodiversity [47,48,49].

The migration analysis using BayesAss (version 3.0.5.6) further supports the presence of strong genetic structures and limited connectivity among populations. Most populations showed high self-recruitment rates, reflecting limited migration and strong genetic stability. For instance, PM and ISL exhibited the highest self-recruitment rates (94.24% and 90.95%, respectively), highlighting their isolation and genetic integrity. In contrast, NT showed the lowest self-recruitment rate (84.63%) and higher levels of incoming migration from NC and NW, with migration rates of 2.50% and 2.60%, respectively. These findings suggest that NT serves as a genetic bridge, facilitating limited connectivity between populations. Additional migration events include NC to NW (1.87%) and NC to NT (1.85%), highlighting the roles of these populations in maintaining genetic exchange within the network [46].

Overall, these analyses suggested a highly structured population system influenced by barriers to gene flow and localized migration patterns. The identified barriers and migration trends likely reflect a combination of historical isolation, environmental heterogeneity, and anthropogenic influences. Populations PM and ISL, with high self-recruitment rates, may require focused conservation efforts to preserve their genetic integrity and minimize the risks of genetic drift or inbreeding. Meanwhile, populations like NT and NW, which act as potential genetic bridges, warrant further investigation to better understand their roles in maintaining connectivity and genetic diversity.

The genetic diversity at the population level in *Salix kusanoi* is low (*A_E_* = 3.852, *H_O_* = 0.222 on average). The least diversity was observed for the NW and NT populations. Previous research has demonstrated that the loss of genetic diversity in isolated populations of *S. phylicifolia* and *S. hukaoana* [34,50] elevates the extinction risk of already endangered species. As *S. kusanoi* reproduces via pollen exchange, the genetic rescue method, or the migration of new individuals from other natural populations into the threatened populations, might help to increase genetic diversity in the concerned populations [51,52]. This method could be applied to the NW and NT populations from Nantou to maintain and conserve genetic diversity.

In the meantime, to conserve the unique SBF population, in situ conservation methods should be applied to maintain its unique genetic variation. The SBF population exhibited high self-recruitment (87.43%) and minimal genetic exchange with other populations, as supported by both the migration and barrier analyses. These analyses also highlighted strong genetic barriers surrounding the SBF population, emphasizing its isolation and distinct genetic structure. In situ conservation should be prioritized for the SBF population as it is an undisturbed natural population. The Shuanglianpi floating island can be monitored as a genetic conservation area with the explicit aim of maintaining population diversity. This method has been applied successfully before in the conservation of wild genetic resources, as establishing genetic conservation areas is a pivotal strategy for preserving the genetic diversity of plant species in their natural habitats. [53].

As *S. kusanoi* can be divided into five main genetic clusters, all populations should be included in the conservation strategy to retain the greatest extent of the species’ evolutionary potential.

## 4. Materials and Methods

### 4.1. Sampling

**Figure 5:**
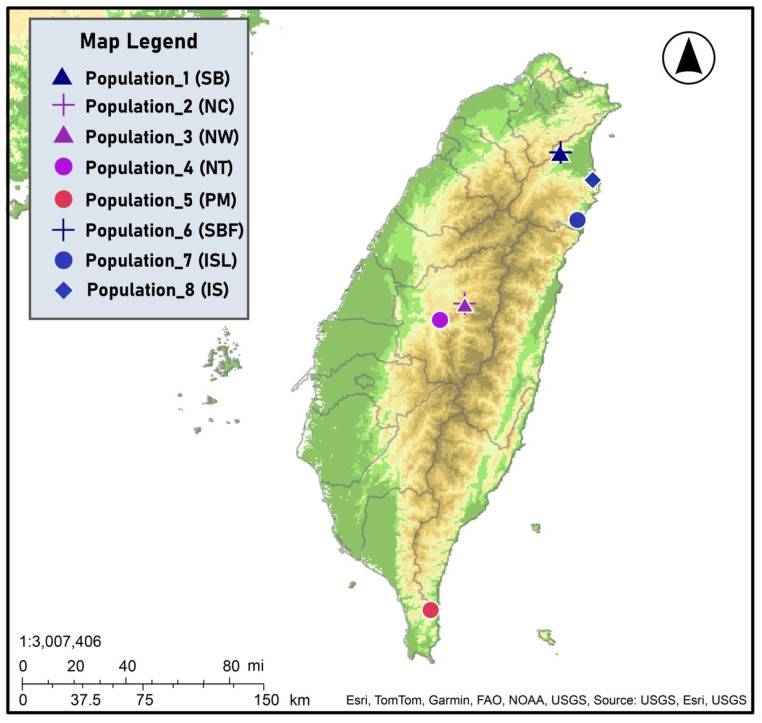
Map of Taiwan with sampling locations for *Salix kusanoi* individuals collected in present study.

We sampled one artificial and seven remaining natural planting populations of *S. kusanoi* across Taiwan, categorizing the distribution into three main regions: northeastern, central, and southern Taiwan. To ensure sample independence, we maintained a minimum distance of 10 m between sampled individuals. In total, we collected 110 individuals from various populations. In northeastern Taiwan, we sampled four populations from Yilan: Shuanglianpi Pond (SB, 20 individuals), Floating Island at Shuanglianpi Pond (SBF, 12 individuals), Sheng Lake (IS, 9 individuals), and Mysterious Lake (ISL, 15 individuals). In central Taiwan, we sampled three populations from Nantou: Zhongming (NC, 11 individuals), Wujie Reservoir (NW, 9 individuals), and Toushe Basin (NT, 6 individuals). In southern Taiwan, we sampled one population from Dongyuan Wetland in Pingtung (PM, 28 individuals) (Table 4, Figure 5). All individuals from SB, SBF, ISL. and IS populations were sampled while a representative group of individuals were sampled from NC, NW, NT, and PM populations. In Toushe Basin, only 20 male old trees remain. To avoid excessive proximity, the sampling distance was approximately 5 m, resulting in a total of 6 individual trees. Sheng Lake, due to the surrounding area being developed into a community, now retains only 9 old trees preserved around the lake. At Wujie Reservoir, being within the reservoir area and with overly dense growth, the selection of old trees for sampling resulted in only 9 individual trees. The SB population is a cultivated population with limited information about its natural origins.

### 4.2. DNA Extraction and Microsatellite Amplification

Total genomic DNA was extracted from dried leaf samples of *S. kusanoi* using a modified method of [54]. The extracted DNA was dissolved in 200 μL of Tris-EDTA (TE) buffer and stored at −20 °C. To evaluate the quality of the extracted genomic DNA, 1% agarose gel electrophoresis was used, with the Lambda marker (200 μg/mL, Promega, Madison, WI, USA) serving as the reference standard. The DNA was then diluted to a concentration of 10 ng/μL in TE buffer, which was used as the template for subsequent experiments.

To investigate the genetic structure of sampled populations, a microsatellite-enriched library of *S. kusanoi* was first constructed and screened using a magnetic bead-based procedure, following an established laboratory protocol [55]. This process yielded 41 potential microsatellite loci. Primers for these loci were then designed based on tandem repeat regions identified using Tandem Repeats Finder (version 4.09) [56] and further optimized for amplification using Primer3 [57]. Out of the 41 loci, 33 were successfully amplified and exhibited polymorphism in a subset of 30 samples tested. Therefore, polymorphisms of the 33 loci were further examined in all samples to elucidate population structure of *S. kusanoi* (Supplementary Table S2).

PCR reactions were carried out using the Labnet MultiGene 96-well Gradient Thermal Cycler (Labnet, Edison, NJ, USA). The thermal cycling conditions included an initial denaturation at 94 °C for 2 min, followed by 35–40 cycles of denaturation at 94 °C for 45 s, annealing at 52–60 °C for 1 min, and extension at 72 °C for 30 s, with a final extension step at 72 °C for 7 min. Microsatellite amplification product genotyping followed the protocol described by [58].

### 4.3. Genetic Diversity

Genetic diversity across eight populations of *S. kusanoi* was assessed using 32 polymorphic microsatellite loci. For each locus, the number of alleles (*A*), effective alleles (*A_E_*), observed heterozygosity (*H_O_*), and expected heterozygosity (*H_E_*) were calculated using GenAlEx 6.5 [59].

Hardy–Weinberg equilibrium (*HWE*) was tested for each locus in all populations using GenAlEx6.5. *F*-statistics, including *F_IS_* (inbreeding coefficient), *F_IT_* (overall inbreeding), and *F_ST_* (population differentiation), were also computed using GenAlEx 6.5 [59].

### 4.4. Genetic Differentiation and Genetic Groups Analysis

Analysis of Molecular Variance (AMOVA), to partition genetic variation at different hierarchical levels, was performed using GenAlEx 6.5 [59]. AMOVA (Analysis of Molecular Variance) was performed to partition genetic variation at 2 levels. First, genetic variation was analyzed across different geographical regions, followed by examination of genetic variation between populations. AMOVA was conducted using GenAlEx 6.5 [59]. A Principal Coordinates Analysis (PCoA) was conducted using GenAlEx 6.5 to visualize the genetic relationships among populations based on the microsatellite data. The first three principal coordinate axes, explaining the largest proportion of genetic variance, were used to infer population clustering and differentiation patterns.

The genetic structure of the populations was inferred using the Bayesian clustering method in STRUCTURE version 2.3.4 [60]. The STRUCTURE analysis was run with 5,000,000 iterations and a burn-in period of 2,000,000 iterations, using the admixture model with correlated allele frequencies. To ensure robust results, the analysis was repeated 10 times, with different random seed initializations to assess the consistency of the results. The optimal number of clusters (*K*) was determined by evaluating delta *K* values and visualizing the results using Structure selector [61]. The plots generated are used to illustrate the proportion of ancestry for each individual and the clustering patterns across populations. The optimal number of genetic clusters (*K*) was determined using the Evanno’s Δ*K* method in Structure selector [35,61]. The genetic relationships among the eight populations of *Salix kusanoi* were analyzed using a Neighbor-Joining (NJ) method based on Nei’s genetic distance. Pairwise genetic distances were calculated following Nei’s formula [62] using the genotype data from 33 SSR loci. The NJ dendrogram was constructed using the “ape” package in R (version 5.8-1) [63].

### 4.5. Migration and Barrier Analysis

Genetic boundaries among georeferenced populations were identified using Monmonier’s Algorithm, implemented through the adegenet package in R [64]. This method detects regions of maximum genetic differentiation within a continuous Voronoi tessellation to determine genetic barriers. Migration rates between populations were estimated using a Bayesian framework in BayesAss 3.0.5 [65]. This analysis is based on Markov chain Monte Carlo techniques and run consisted of 1 × 10^6^ iterations and the first 1 × 10^4^ steps were discarded as burn-in. For these two analyses, the SB population was excluded because it is an artificially established population and does not represent a natural genetic structure, which could bias the results.

## 5. Conclusions

Genetic diversity and the population structure of eight populations of *S. kusanoi* in Taiwan were assessed using 33 microsatellites in this study. The genetic diversity in *Salix kusanoi* was low, but among the eight populations studied, the SBF population exhibited unique characteristics of genetic admixture. Among the eight populations, five distinct genetic clusters were observed, and the clustering pattern could also be attributed to their geographical location. These results may reflect their evolutionary history and geographical isolation. Anthropogenic activities may also contribute to the loss of genetic diversity. As further loss of genetic diversity is harmful to the survival of this species, we recommend genetic rescue, ex situ conservation, and in situ conservation as strategies to conserve *S. kusanoi*. Special emphasis should be placed on in situ conservation for the SBF population to maintain its unique genetic variation and protect it as a natural genetic conservation area. Additionally, genetic rescue is critical for the NW and NT populations to ensure their viability. These conservation strategies, when combined with habitat protection and ongoing monitoring, will help preserve the genetic diversity and evolutionary potential of *S. kusanoi*.

## Figures and Tables

**Table 1:** Molecular variance analysis (AMOVA) of *Salix kusanoi* (Hayata) across geographical regions using 33 microsatellite loci. *: *p* < 0.05; d.f.: degree of freedom; and S.S. sum of square.

Source	d.f.	S.S.	Mean S.S.	Estimated Variance	%	Fixation Index
Across geographic regions						
Among regions	2	238.29	119.15	1.54	15	*F_ST_* = 0.15 *
Among populations	107	1479.57	13.83	5.08	49	*F_IS_* = 0.58 *
Within populations	110	402.50	3.66	3.66	36	*F_IT_* = 0.64 *
Total	219	2120.36		10.29	100	

**Table 2:** Nei’s genetic distance among 8 populations.

Populations	SBF	ISL	IS	SB	NC	NW	NT	PM
SBF	-------	0.5773	0.6442	0.6682	0.6435	0.6873	0.6238	0.6818
ISL	0.5773	-------	0.5782	0.7509	0.6802	0.7013	0.6637	0.7047
IS	0.6442	0.5782	-------	0.6783	0.6616	0.7399	0.6702	0.7315
SB	0.6682	0.7509	0.6783	-------	0.6062	0.6656	0.5868	0.6714
NC	0.6435	0.6802	0.6616	0.6062	-------	0.5336	0.4779	0.6014
NW	0.6873	0.7013	0.7399	0.6656	0.5336	-------	0.4174	0.6342
NT	0.6238	0.6637	0.6702	0.5868	0.4779	0.4174	-------	0.6161
PM	0.6818	0.7047	0.7315	0.6714	0.6014	0.6342	0.6161	-------

**Table 3:** The migration matrix of the 7 populations (the values in parentheses represent the uncertainty or variability in the migration rate estimate).

Populations	To NC	To NW	To NT	To PM	To SBF	To ISL	To IS
From NC	0.8885 (0.0363)	0.0187 (0.0177)	0.0185 (0.0175)	0.0179 (0.0170)	0.0191 (0.0182)	0.0180 (0.0168)	0.0192 (0.0181)
From NW	0.0382 (0.0268)	0.8575 (0.0409)	0.0207 (0.0194)	0.0203 (0.0186)	0.0208 (0.0197)	0.0212 (0.0205)	0.0213 (0.0198)
From NT	0.0250 (0.0228)	0.0260 (0.0239)	0.8463 (0.0446)	0.0256 (0.0235)	0.0262 (0.0242)	0.0259 (0.0240)	0.0251 (0.0230)
From PM	0.0097 (0.0093)	0.0094 (0.0090)	0.0094 (0.0094)	0.9424 (0.0209)	0.0098 (0.0095)	0.0098 (0.0092)	0.0095 (0.0094)
From SBF	0.0174 (0.0162)	0.0173 (0.0168)	0.0174 (0.0164)	0.0177 (0.0163)	0.8951 (0.0346)	0.0180 (0.0171)	0.0171 (0.0162)
From ISL	0.0151 (0.0141)	0.0148 (0.0144)	0.0151 (0.0143)	0.0151 (0.0145)	0.0152 (0.0145)	0.9095 (0.0307)	0.0152 (0.0145)
From IS	0.0205 (0.0192)	0.0213 (0.0203)	0.0215 (0.0199)	0.0212 (0.0202)	0.0205 (0.0193)	0.0207 (0.0193)	0.8743 (0.0395)

**Table 4:** Sample collection of *Salix kusanoi*. NE: northeast; C: central; and S: southern regions of Taiwan.

Populations	Geographic Locations	Symbols	No. of Individuals
(1) Shuanglian Pond	Yilan County (NE)	SB	20
(2) Zhongming Village, Yuchi Township	Nantou County (C)	NC	11
(3) Wujie Reservoir, Ren’ai Township	Nantou County (C)	NW	9
(4) Toushe Basin	Nantou County (C)	NT	6
(5) Dongyuan Wetlands, Mudan Township	Pingtung County (S)	PM	28
(6) Floating Island at Shuanglian Pond	Yilan County (NE)	SBF	12
(7) Mysterious Lake, Nanao Township	Yilan County (NE)	ISL	15
(8) Sheng Lake, Su’ao Township	Yilan County (NE)	IS	9
Total			110

## Data Availability

The original contributions in the study are included in the Supplementary Material as raw data sheets. Further inquiries can be directed to the corresponding author.

## Supplementary Materials

The following supporting information can be downloaded at https://www.mdpi.com/article/10.3390/plants14071080/s1: Table S1: Raw data of 110 samples from *Salix kusanoi* populations in Taiwan based on amplicon sizes using 33 SSR loci; Table S2: Specific primer sequences, basic motif units, number of genotypes, annealing temperature, and expected fragment sizes for 41 microsatellite loci; Table S3: Genetic diversity indices of *Salix kusanoi* (Hayata) in Taiwan, analyzed using 33 microsatellite loci. Table S4: Population information of the sampled individuals.

## References

[1] Nic Lughadha E., Bachman S.P., Leão T.C., Forest F., Halley J.M., Moat J., Acedo C., Bacon K.L., Brewer R.F., Gâteblé G. (2020). Extinction Risk and Threats to Plants and Fungi. Plants People Planet.

[2] Arciszewski M., Pogorzelec M., Parzymies M., Bronowicka-Mielniczuk U., Mieczan T. (2024). Do Endangered Glacial Relicts Have a Chance for Effective Conservation in the Age of Global Warming? A Case Study: *Salix lapponum* in Eastern Poland. Biology.

[3] Pimm S.L., Raven P. (2000). Extinction by Numbers. Nature.

[4] Gramlich S., Sagmeister P., Dullinger S., Hadacek F., Hörandl E. (2016). Evolution In Situ: Hybrid Origin and Establishment of Willows (*Salix* L.) on Alpine Glacier Forefields. Heredity.

[5] Mitsch W.J., Gosselink J.G. (2000). The Value of Wetlands: Importance of Scale and Landscape Setting. Ecol. Econ..

[6] Díaz-Alba D., Henry A.L., de Jalón D.G., del Tánago M.G., Martínez-Fernández V. (2023). *Salix* Regeneration in Fluvial Landscapes: Empirical Findings Based on a Systematic Review. Ecol. Eng..

[7] Richardson J., Isebrands J.G. Poplars and willows: Trees for society and the environment. Proceedings of the 13th North American Agroforestry Conference.

[8] Grandstaff G., Kuzovkina Y.A., Legrand A. (2023). Attraction of Bees to Native and Introduced Willows (*Salix* spp.). Forests.

[9] Huang C.L., Chang C.T., Huang B.H., Chung J.D., Chen J.H., Chiang Y.C., Hwang S.Y. (2015). Genetic Relationships and Ecological Divergence in Salix Species and Populations in Taiwan. Tree Genet. Genomes.

[10] Yang K.C., Huang T.C. (1996). Flora of Taiwan.

[11] Editorial Committee of the Red List of Vascular Plants of Taiwan (2017). The Red List of Vascular Plants of Taiwan.

[12] Lu S.Y., Pan F.J. (1998). *Salix kusanoi*. The IUCN Red List of Threatened Species. https://www.iucnredlist.org/species/31250/9619786.

[13] Hughes A.R., Inouye B.D., Johnson M.T., Underwood N., Vellend M. (2008). Ecological Consequences of Genetic Diversity. Ecol. Lett..

[14] Bloju O., Ihos S.C., Rus A.B., Ciaparii R., Popescu S. (2017). Comparative Analysis of Different Molecular Markers Used for Evaluation of *Salix* sp. Variability. J. Hortic. For. Biotechnol..

[15] Sharma J.P., Thakur S., Sharma P., Thakur A. (2024). Genetic Structure and Gene Flow among Populations of Willow (*Salix* Species). Ecol. Genet. Genom..

[16] Chen Y., Wang T., Fang L., Li X., Yin T. (2016). Confirmation of Single-Locus Sex Determination and Female Heterogamety in Willow Based on Linkage Analysis. PLoS ONE.

[17] Cortés A.J., Waeber S., Lexer C., Sedlacek J., Wheeler J.A., van Kleunen M., Boßdorf O., Hoch G., Rixen C., Wipf S. (2014). Small-Scale Patterns in Snowmelt Timing Affect Gene Flow and the Distribution of Genetic Diversity in the Alpine Dwarf Shrub *Salix herbacea*. Heredity.

[18] He X., Zheng J., Zhou J., He K., Shi S., Wang B. (2015). Characterization and Comparison of EST-SSRs in Salix, Populus, and Eucalyptus. Tree Genet. Genomes.

[19] Hernández-Leal M.S., Suárez-Atilano M., Piñero D., González-Rodríguez A. (2019). Regional Patterns of Genetic Structure and Environmental Differentiation in Willow Populations (*Salix humboldtiana* Willd.) from Central Mexico. Ecol. Evol..

[20] Hao L., Zhang G., Lu D., Hu J., Jia H. (2019). Analysis of the Genetic Diversity and Population Structure of *Salix psammophila* Based on Phenotypic Traits and Simple Sequence Repeat Markers. PeerJ.

[21] Hu J.J., Lv J.H., Lu M.Z. (2011). Genetic Linkage Map of Willow (*Salix leucopithecia* × *S. erioclada* L.) Based on AFLP and SSR Markers. BMC Proc..

[22] Kim Y.Y., Kwon S.H., Jo A., Kim Y.G., Lee J.W., Kang K.S. (2018). Single Nucleotide Polymorphism (SNP) Characterization of Drought-Responsive Genes to Estimate Genetic Variation of *Populus tremula* var. davidiana and Eight Other *Populus* Species. Can. J. For. Res..

[23] Perdereau A.C., Kelleher C.T., Douglas G.C., Hodkinson T.R. (2014). High Levels of Gene Flow and Genetic Diversity in Irish Populations of *Salix caprea* L. Inferred from Chloroplast and Nuclear SSR Markers. BMC Plant Biol..

[24] Tsarouhas V., Gullberg U., Lagercrantz U. (2002). An AFLP and RFLP Linkage Map and Quantitative Trait Locus (QTL) Analysis of Growth Traits in *Salix*. Theor. Appl. Genet..

[25] Barker J.H.A., Pahlich A., Trybush S., Edwards K.J., Karp A. (2003). Microsatellite Markers for Diverse *Salix* Species. Mol. Ecol. Notes.

[26] Bozzi J.A., Liepelt S., Ohneiser S., Gallo L.A., Marchelli P., Leyer I., Ziegenhagen B., Mengel C. (2015). Characterization of 23 Polymorphic SSR Markers in *Salix humboldtiana* (Salicaceae) Using Next-Generation Sequencing and Cross-Amplification from Related Species. Appl. Plant Sci..

[27] Carletti G., Cattivelli L., Vietto L., Nervo G. (2021). Multiallelic and Multilocus Simple Sequence Repeats (SSRs) to Assess the Genetic Diversity of a *Salix* spp. Germplasm Collection. J. For. Res..

[28] Değirmenci F.Ö., Çiftçi A., Acar P., Kaya Z. (2021). Genetic Diversity and Population Structure of *Salix alba* Across River Systems in Turkey and Their Importance in Conservation Management. Plant Ecol. Divers..

[29] Hanley S., Barker J., Van Ooijen J., Aldam C., Harris S., Åhman I., Larsson S., Karp A. (2002). A Genetic Linkage Map of Willow (*Salix viminalis*) Based on AFLP and Microsatellite Markers. Theor. Appl. Genet..

[30] Jia H., Yang H., Sun P., Li J., Zhang J., Guo Y., Han X., Zhang G., Lu M., Hu J. (2016). De Novo Transcriptome Assembly, Development of EST-SSR Markers and Population Genetic Analyses for the Desert Biomass Willow, *Salix psammophila*. Sci. Rep..

[31] Nagamitsu T., Hoshikawa T., Kawahara T., Barkalov V.Y., Sabirov R.N. (2014). Phylogeography and Genetic Structure of Disjunct *Salix arbutifolia* Populations in Japan. Popul. Ecol..

[32] Reisch C., Schurm S., Poschlod P. (2007). Spatial Genetic Structure and Clonal Diversity in an Alpine Population of *Salix herbacea* (Salicaceae). Ann. Bot..

[33] Tokdemir Y., Değirmenci F.Ö., Uluğ A., Acar P., Kaya Z. (2024). Genetic Diversity of *Salix caprea* L. Populations in Fragmented Habitats of Northeastern Türkiye. Biologia.

[34] Kikuchi S., Suzuki W., Sashimura N. (2011). Gene Flow in an Endangered Willow *Salix hukaoana* (Salicaceae) in Natural and Fragmented Riparian Landscapes. Conserv. Genet..

[35] Evanno G., Regnaut S., Goudet J. (2005). Detecting the Number of Clusters of Individuals Using the Software STRUCTURE: A Simulation Study. Mol. Ecol..

[36] Zhai F.F., Liu J.X., Li Z.J., Mao J.M., Qian Y.Q., Han L., Sun Z.Y. (2017). Assessing Genetic Diversity and Population Structure of *Salix viminalis* Across Ergun and West Liao Basin. Silva Fenn..

[37] Lin J., Gibbs J.P., Smart L.B. (2009). Population Genetic Structure of Native versus Naturalized Sympatric Shrub Willows (*Salix*; Salicaceae). Am. J. Bot..

[38] Trybush S.O., Jahodová Š., Čížková L., Karp A., Hanley S.J. (2012). High Levels of Genetic Diversity in Salix viminalis of the Czech Republic as Revealed by Microsatellite Markers. BioEnergy Res..

[39] Stamati K., Hollingsworth P.M., Russell J.J.P.S. (2007). Patterns of Clonal Diversity in Three Species of Sub-Arctic Willow (*Salix lanata*, *Salix lapponum*, and *Salix herbacea*). Plant Syst. Evol..

[40] Sochor M., Vašut R.J., Bártová E., Majeský Ľ., Mráček J. (2013). Can Gene Flow Among Populations Counteract the Habitat Loss of Extremely Fragile Biotopes? An Example from the Population Genetic Structure in *Salix daphnoides*. Tree Genet. Genomes.

[41] Castiglione S., Cicatelli A., Lupi R., Patrignani G., Fossati T., Brundu G. (2010). Genetic Structure and Introgression in Riparian Populations of *Populus alba* L. Plant Biosyst..

[42] Lee K.M., Kim Y.Y., Hyun J.O. (2011). Genetic Variation in Populations of *Populus davidiana* Dode Based on Microsatellite Marker Analysis. Genes Genom..

[43] Wright S. (1978). Evolution and the Genetics of Populations. IV. Variability Within and Among Natural Populations.

[44] Smulders M.J.M., Cottrell J.E., Lefèvre F., Van der Schoot J., Arens P., Vosman B., Tabbener H.E., Grassi F., Fossati T., Castiglione S. (2008). Structure of the Genetic Diversity in Black Poplar (*Populus nigra* L.) Populations Across European River Systems: Consequences for Conservation and Restoration. For. Ecol. Manag..

[45] Callahan C.M., Rowe C.A., Ryel R.J., Shaw J.D., Madritch M.D., Mock K.E. (2013). Continental-Scale Assessment of Genetic Diversity and Population Structure in Quaking Aspen (*Populus tremuloides*). J. Biogeogr..

[46] Zhai F., Mao J., Liu J., Peng X., Han L., Sun Z. (2016). Male and Female Subpopulations of *Salix viminalis* Present High Genetic Diversity and High Long-Term Migration Rates Between Them. Front. Plant Sci..

[47] Manni F., Guérard E., Heyer E. (2004). Geographic Patterns of (Genetic, Morphologic, Linguistic) Variation: How Barriers Can Be Detected by Using Monmonier’s Algorithm. Hum. Biol..

[48] Patten M.A., Smith-Patten B.D. (2008). Biogeographical Boundaries and Monmonier’s Algorithm: A Case Study in the Northern Neotropics. J. Biogeogr..

[49] Li C., Yang S., Zhao J.X., Dosseto A., Bi L., Clark T.R. (2016). The Time Scale of River Sediment Source-to-Sink Processes in East Asia. Chem. Geol..

[50] Egelund B., Pertoldi C., Barfod A.S. (2012). Isolation and Reduced Gene Flow Among Faroese Populations of Tea-Leaved Willow (*Salix phylicifolia*, Salicaceae). New J. Bot..

[51] Ingvarsson P.K. (2001). Restoration of Genetic Variation Lost—The Genetic Rescue Hypothesis. Trends Ecol. Evol..

[52] Tallmon D.A., Luikart G., Waples R.S. (2004). The Alluring Simplicity and Complex Reality of Genetic Rescue. Trends Ecol. Evol..

[53] National Research Council (1993). Managing Global Genetic Resources: Agricultural Crop Issues and Policies.

[54] Doyle J.J., Dickson E.E. (1987). Preservation of Plant Samples for DNA Restriction Endonuclease Analysis. Taxon.

[55] Lu H.Y., Shih H.C., Ju L.P., Hwang C.C., Chiang Y.C. (2018). Characterization of 39 Microsatellite Markers from *Nuphar shimadai* (Nymphaeaceae) and Cross-Amplification in Two Related Taxa. Appl. Plant Sci..

[56] Benson G. (1999). Tandem Repeats Finder: A Program to Analyze DNA Sequences. Nucleic Acids Res..

[57] Rozen S., Skaletsky H. (1999). Primer3 on the WWW for General Users and for Biologist Programmers. Bioinformatics Methods and Protocols.

[58] Mantiquilla J.A., Shiao M.S., Lu H.Y., Sridith K., Sidique S.N.M., Liyanage W.K., Chu Y.L., Shih H.C., Chiang Y.C. (2022). Deep Structured Populations of Geographically Isolated Nipa (*Nypa fruticans* Wurmb.) in the Indo-West Pacific Revealed Using Microsatellite Markers. Front. Plant Sci..

[59] Peakall R., Smouse P.E. (2012). GenAlEx 6.5: Genetic Analysis in Excel. Population Genetic Software for Teaching and Research—An Update. Bioinformatics.

[60] Pritchard J.K., Wen W., Falush D. (2010). Documentation for Structure Software.

[61] Li Y.L., Liu J.X. (2018). StructureSelector: A Web-Based Software to Select and Visualize the Optimal Number of Clusters Using Multiple Methods. Mol. Ecol. Resour..

[62] Nei M. (1972). Genetic Distance Between Populations. Am. Nat..

[63] Paradis E., Schliep K. (2019). ape 5.0: An Environment for Modern Phylogenetics and Evolutionary Analyses in R. Bioinformatics.

[64] Jombart T. (2008). adegenet: An R Package for the Multivariate Analysis of Genetic Markers. Bioinformatics.

[65] Wilson G.A., Rannala B. (2003). Bayesian Inference of Recent Migration Rates Using Multilocus Genotypes. Genetics.

